# Application of High-Resolution Infrared Thermography to Study the Effects of Technologically Processed Antibodies on the Near-Surface Layer of Aqueous Solutions

**DOI:** 10.3390/molecules29184309

**Published:** 2024-09-11

**Authors:** Elena Don, Evgenii Zubkov, Ekaterina Moroshkina, Irina Molodtsova, Anastasia Petrova, Sergey Tarasov

**Affiliations:** 1 R&D Department, OOO “NPF “Materia Medica Holding”, 47-1, Trifonovskaya St., 129272 Moscow, Russia; zubkovev@materiamedica.ru (E.Z.); moroshkinaes@materiamedica.ru (E.M.); molodcovaiv@materiamedica.ru (I.M.); petrovaao@materiamedica.ru (A.P.); tarasovsa@materiamedica.ru (S.T.); 2 Laboratory of Physiologically Active Substances, Federal State Budgetary Scientific Institute of General Pathology and Pathophysiology, 8, Baltiyskaya St., 125315 Moscow, Russia

**Keywords:** IR thermography, technologically processed antibodies, quality control, aqueous solutions

## Abstract

A new class of biologics is obtained using the technologically processed of antibodies (TPA), which are used as the initial substance, and their dilution at each stage is accompanied by a controlled external vibrational (mechanical) treatment. This article focuses on the development and validation of a novel technique that can be applied for assessing the identity of TPA-based drugs. It has previously been found that after such treatment, the resulting solution either acquired new properties that were not present in the initial substance or a quantitative change in properties compared to the initial substance was observed. The use of mechanical treatment during the manufacture of the TPA-based drugs can cause the formation of new bonds between the solvent and antibody molecules. These changes manifest themselves in altered adsorption at the surface of the test solutions, which results in the formation of a near-surface film. One of the indicators of such events is the change in the surface temperature of the solution, which can be analyzed using high-resolution thermography. Unlike other methods, the high-resolution thermography allows the near-surface layer of a heterogeneous aqueous solution to be clearly visualized and quantified. A number of experiments were performed: seven replicates of sample preparations were tested; the influence of factors “day” or “operator” was investigated during 12 days of testing by two operators. The method also allowed us to distinguish between technologically processed antibodies and samples containing technologically processed buffer. The thermographic analysis has proven to be a simple, specific, and reproducible technique that can be used to analyze the identity of TPA-based drugs, regardless of the dosage form tested.

## 1. Introduction

New drug products, “gradualised” drugs, are based on technologically processed antibodies (TPA) manufactured using the technology of gradual dilution of the initial pharmaceutical substance combined with a standardized external rhythmic (mechanical) effect [1]. When applying this technology, the decrease in the concentration of the initial substance is quite significant, exceeding 10^24^ times. At present, direct analytical techniques for the identification of the initial substance in such dilutions are not available. To ensure the finished “gradualised” drugs have the required identity, a quality control spectrophotometric method based on the assessment of the TPA effect on the oxidation of ascorbic acid in solution [2] has been previously developed. However, the excipients contained in some dosage forms may affect the oxidation of ascorbic acid, preventing identification. Thus, new quality control methods based on a different approach are required.

During the research on TPA, besides their safety and effectiveness for various health conditions, it has been found that they exert a modifying effect, which is specific, i.e., aimed at the biological target of the corresponding antibodies [3]. In addition to the specific activity, the ability of TPA to alter the structure of a neutral medium (water, water-alcohol mixture, lactose, or semi-crystalline medium) has been discussed [4,5,6,7]. For example, it has been shown that when highly diluted aqueous solutions are added to water, its hydrogen bonds change [8,9,10]. Using low-frequency Raman spectroscopy and time-resolved terahertz spectroscopy, the difference has been shown in the oscillation parameters of the hydrogen bonds between samples containing TPA and the respective controls [11]. The reason for the breaking of intermolecular and intramolecular bonds [12], resulting in the emergence of new bonds with molecules of a substance used as an active pharmaceutical ingredient, should be the process of exerting an external mechanical effect (one of the main stages of producing drugs based on TPA) [13,14]. 

Detecting changes induced by mechanical effect is a complex task. Some physical-chemical properties of solutions containing TPA differ from those detected for control solutions: pH, electrical conductivity, the content of reactive oxygen species, and free radicals, etc. [9,15,16,17]. However, any changes in the properties of aqueous solutions eventually result in changes in the properties of water at the air–liquid interface.

The physical-chemical properties of a liquid surface are closely related to its volume. The phenomena of convection, diffusion, adsorption and desorption lead to heat and mass transfer between the surface and volume. The study of the surface properties of a liquid, in particular the determination of the concentration of surface excess, is carried out using the dependence of the surface tension coefficient on temperature. For this purpose the Gibbs formula, derived from thermodynamics, is used. The Gibbs formula describes the equilibrium state of a surface with an excess surface concentration, which depends on temperature. According to the Gibbs approach, the properties of the near-surface layer are described by adsorption from the bulk phase on the surface (which, in general, can be described by Equation (1)).

A = −(1/RT) × (dn/ln dC)(1)

where A is adsorption (in relative units), R is the Boltzmann constant, T is absolute temperature, n characterizes surface tension, and C is concentration [18].

In this case, adsorption is an equilibrium value characterized by the ratio of the dynamic constants of adsorption and desorption of a substance from the bulk phase to the surface and back.

Since the technological processing used to prepare the active pharmaceutical ingredient of “gradualized drugs” leads to a sequence of events associated with a sharp redistribution of the structure of aqueous solutions and the formation of new bonds between the substance molecules (antibody) [1], changes in the properties of the test solutions should affect one or more variables of the Gibbs Equation (1). These variables, in turn, can be detected using high-resolution thermography and evidenced by changes in adsorption on the surface of the test solutions (i.e., in the formation of a near-surface film that can be detected quantitatively). On thermograms, the concentration of surface excess is visualized as a darker area (with a temperature approximately 0.5–1 °C lower than the temperature of the surrounding sample). In the image obtained from the IR camera, the surface area of the entire vessel with sample (e.g., Petri dish) is taken conditionally to be 1. It is important to highlight that in further mathematical calculations, any of the values can be operated on the proportion of the film area or the proportion of the area free of the film, which in total are equal to 1.

The above-mentioned facts formed the basis for the development of a test system for identification of drugs based on TPA. The technique of high-resolution IR thermography is widely used for qualitative and quantitative determination of impurity concentrations in aqueous solutions [19]. It is important to note that the high-resolution IR thermography method is characterized by simplicity and clarity due to the absence of multistage transformations associated with chemical transformations and the absence of the need to use special indicator compounds. For some dosage forms, this was much more convenient compared to the kinetic method used for identification of TPA [2]. As for other approaches that allow the influence of TPA’s on the physical-chemical properties of solutions to be determined, whether it is low-frequency Raman spectroscopy or time-resolved terahertz spectroscopy, their application turns out to be more time-consuming and expensive during routine measurements. Since high-resolution thermography allows tracking the rate of changes in adsorption on the surface of the test solutions, this method evaluates the changes in the composition of the TPA solution compared to the control.

For validation of the identification method, only evaluation of the specificity of the analysis is required [20]. Additionally, we assessed repeatability and intermediate precision. The method of thermographic analysis has proven to be a reproducible and specific technique that can be used to analyze TPA-based drugs, regardless of the dosage form tested.

## 2. Results and Discussion

**Figure 1:**
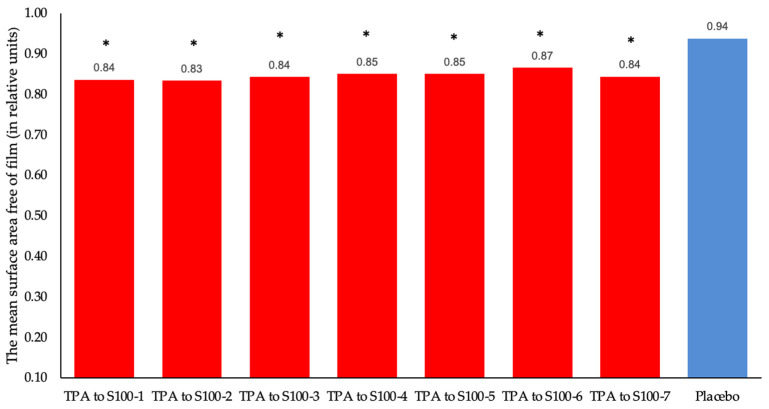
The mean surface area free of film for samples of one batch of the test sample and placebo. (*—*p* < 0.1 vs. placebo, all q > 17.8, qcrit = 3.98, Tukey test).

IR thermography allows us to directly determine change in the structure of water in the presence of a TPA sample, which is assessed based on the change in adsorption on the surface of the studied solutions in comparison with the solution of the control sample. The prepared solutions of the test and control samples are placed in a Petri dish, and the average area of the near-surface film on the surface of the solution and the free of film surface are measured using a thermal imager in a given temperature range. To assess the repeatability, seven replicates of the same test sample were tested (for validation, a minimum of six measurements is required [20]). The results are shown in Figure 1. It can be seen that all seven samples show high repeatability for the method proposed and are statistically significantly different in the mean surface area free of film from the result obtained for the placebo sample (*p* < 0.1 based on the Tukey test) without differing from each other.

**Figure 2:**
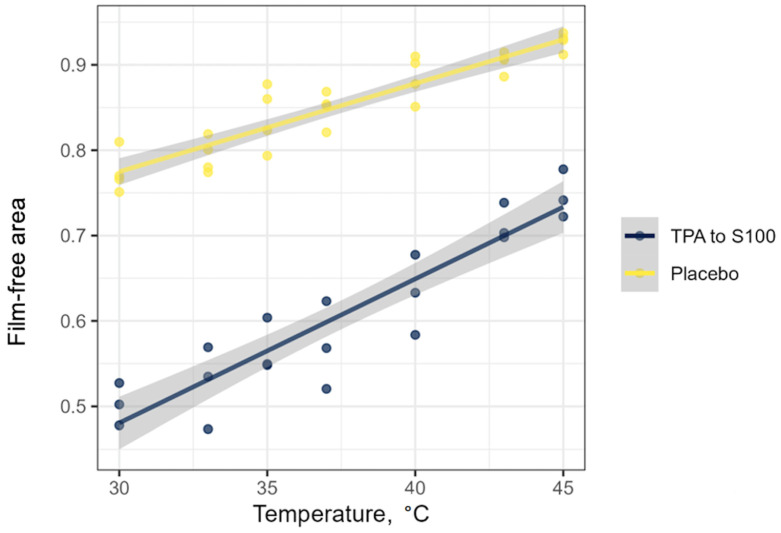
An example of a scatter plot with straight line approximation and its confidence interval.

The study has taken into account various factors that could affect the quality of the analysis and the reproducibility of the data. Intermediate precision, which expresses within-laboratory variations like different days or analysts, was evaluated in a 12 day study examining analytical results obtained by two different operators. As a result of each experiment, the difference coefficient of the near-surface film formation in solution (∆S) was calculated for the test and control samples. As a control sample, a placebo was used, which was prepared using the same technology as for TPA but with a buffer solution instead of the initial substance. Next, the observations at temperatures ranging from 30 to 45 °C were adjusted as described in the “Statistical analysis” section (Figure 2). It is important to highlight that surface area free of film never reaches 100% even for placebo (technologically processed buffer), which differs from pure water. 

**Figure 3:**
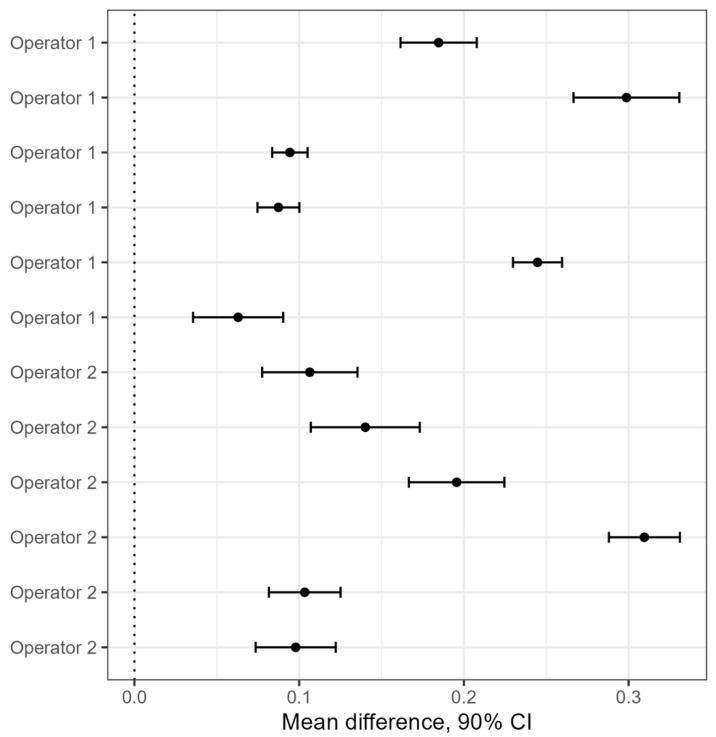
Confidence intervals which show the difference in the mean surface areas free of film for the compared groups of samples.

Figure 3 shows the data with 90% confidence intervals of the difference between the placebo group and the drug group. The inclusion of a confidence interval of “zero” value indicates the absence of statistically significant differences between the groups. As all the confidence intervals excluded the “zero” value, the results of the statistical analysis show a statistically significant difference from placebo in all 12 cases, which is thus not related to the day or operator. Since the methodology was developed as a qualitative one, there was no need to take into account the size of the observed differences; the statistical significance of these differences turns out to be a necessary and sufficient criterion in this case.

**Figure 4:**
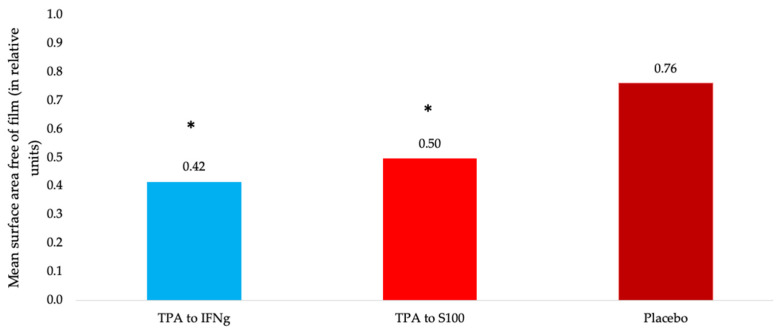
Mean surface area free of film (in relative units) for samples of TPAs to various molecules, and control (*—*p* < 0.1 vs. placebo sample, q1 = 36.5, q2 = −25.5, qcrit = 2.95, Tukey test).

Suitable identification tests should be able to discriminate between compounds of closely related structures that are likely to be present in the samples. To assess the specificity of the technique for the initial antibodies, a number of TPAs to various molecules were tested (Examples of images of a Petri dish with a surface film formed during cooling for all the samples could be fons in Supplementary Materials (Figure S1). Figure 4 shows an example of such an experiment for a sample based on TPA to IFNγ, a sample based on TPA to the S100 calcium binding protein B, and a placebo sample. 

Samples of TPA to IFNγ and TPA to S100B have been shown to be statistically significantly different from the control (placebo), but not from each other. Hence, it can be concluded that this model system at the current stage of development does not allow the technologically processed dilutions of various antibodies to be distinguished from each other, which may be due to the structural similarity between antibodies. 

The main structural unit of immunoglobulins is the dimer of two identical pairs of light and heavy chains (L—H)2 [21,22,23]. The similarity of the structural organization of antibodies can potentially lead to a similarity of the physical-chemical characteristics of various TPAs. Therefore, this technique should be considered qualitative, which allows TPA to be distinguished from controls, and, consequently, it can be used for the identification of TPA solutions. 

The specific determination of a given type of TPA is possible using immunological methods, such as ELISA [24] or immunosensors [25], which are based on detecting the effect of TPA on binding of the corresponding antigen and antibody. However, for the analysis of identity, the developed approach is fully sufficient, simple, does not require additional reagents (as compared to the kinetic assay [2]), and can be easily mastered by new employees. The technique of high-resolution IR thermography is widely used for qualitative and quantitative determination of impurity concentrations in aqueous solutions [19,26] but there are also other methods for studying the air–water interface. For example, shadowgraphy was the first to suggest the complex structure of the surface layer but necessitates a relatively intricate setup of the experimental apparatus, including the formation of thick parallel beams [27]. Particle Image Velocimetry allows assessing the air–water interface but has reduced resolution in the vertical direction near the surface [28]. Laser-induced fluorescence is affected by the concentration gradient of emitting particles in the surface layer, which can be mistakenly interpreted as a temperature gradient [29].

## 3. Materials and Methods

### 3.1. Reagents and Devices

Purified water obtained using a Milli-Q high purity water system (Millipore, Darmstadt, Germany) was used in the study in order to minimize the influence of impurities in water on the formation of near-surface films. Image capture was performed using a VarioCam HD head high-resolution thermography camera (InfraTec, Dresden, Germany) and the IR1.0/30 LW lens (Jenoptik, Jena, Germany). Images were processed with IRBIS 3.1 professional software (InfraTec, Germany). 

Petri dishes used in the study are made of neutral transparent polystyrene. The density is 1.05 g/cm^2^. The surface water absorption is <0.05%. These dishes are chemically resistant to oils, alcohols, bases, fats, salts, boric acid, citric acid, formic acid, hydrofluoric acid, lactic acid, oxalic acid, salicylic acid, tartaric acid, hydrogen peroxide, phenol, potassium permanganate, triethylene and tripropylene glycol, urea.

### 3.2. Test Samples

Affinity purified rabbit polyclonal antibodies to S100 calcium binding protein B (2.5 mg/mL) were manufactured (Ab Biotechnology Limited, Penicuik, UK) in accordance with the GMP requirements for starting materials. TPA to S100 were produced by the GMP-compliant manufacturing facility of OOO “NPF “Materia Medica Holding” in accordance with the technology described in the United States Patent 8535664 [30]. Briefly, technological processing of antibodies to S100B consisted of sequential multiple dilutions followed by controlled intensive hydrodynamic treatment at each dilution step. Briefly, Ab to S100B was mixed with a solvent at a ratio of 1:100 and shaken vigorously with impact by hand with a controlled frequency of about 4 Hz (21 strokes in about 4.8 s). For the preparation of all dilutions (1:100 at each stage), a water-alcohol solution was used, except for the final two dilutions, which were prepared with purified water. The theoretical level of reduction in the concentration of the original antibodies was at least 10^24^ times. Although a number of authors claim that molecules of the original substance can be retained even in high dilutions due to the flotation effect [31,32], the activity of high dilutions is associated not so much with the presence of an insignificant number of the original substance molecules as with the presence of nanoassociates that self-organize during multiple dilutions with shaking [33,34]. The above-described TPA to S100B protein was mixed with purified water, maltitol, glycerol, potassium sorbate, and anhydrous citric acid until a homogeneous solution was obtained. Thus, TENOTEN^®^ FOR CHILDREN (oral drops) was prepared. Anaferon^®^ for children (oral drops) and placebo were prepared in a similar way, but instead of antibodies to S100B, antibodies to interferon-gamma (2.5 mg/mL, Ab Biotechnology Limited, UK) or phosphate-buffered saline (pH 7.2, made from a tablet (Sigma Aldrich, St. Louis, MO, USA)) were used as the initial substance, respectively.

### 3.3. Technique Developed

The study took into account various factors that could affect the quality of the analysis and the reproducibility of the data. In addition, to ensure uniform cooling of the samples, the experiments were carried out at an ambient temperature in the range of 21–23 °C. In order to improve the visualization of films, a temperature range from 30 °C to 50 °C was used. This temperature range was selected experimentally based on data analysis and is the most informative and convenient in the research. And, finally, in order to exclude the influence of plastic on the physical-chemical properties of water, dishes of the same manufacturer were used during one series of experiments. To minimize contamination of samples during analysis, new sterile Petri dishes were used. 

### 3.4. Device Calibration

A thermography camera was turned on and an empty Petri dish (Poliefir, Frunze, Belarus) was placed on the bottom of the measuring box, and the image was focused. Then, the Petri dish was removed, and a sheet of black velvet paper was put on the bottom of the measuring box. The camera was calibrated using a non-uniformity correction (NUC) on a black matte surface. Using the IRBIS 3.1 professional software (InfraTec, Germany), the following parameters for image acquisition were set:

- The number of frames in the file—1500;
- The time interval between frames—1 s;
- Acquisition time—25 min;
- Emissivity—0.98.

The temperature inside the box was measured using a Testo 720 thermometer (Testo, Titisee-Neustadt, Germany).

### 3.5. Device Functionality Testing

**Figure 5:**
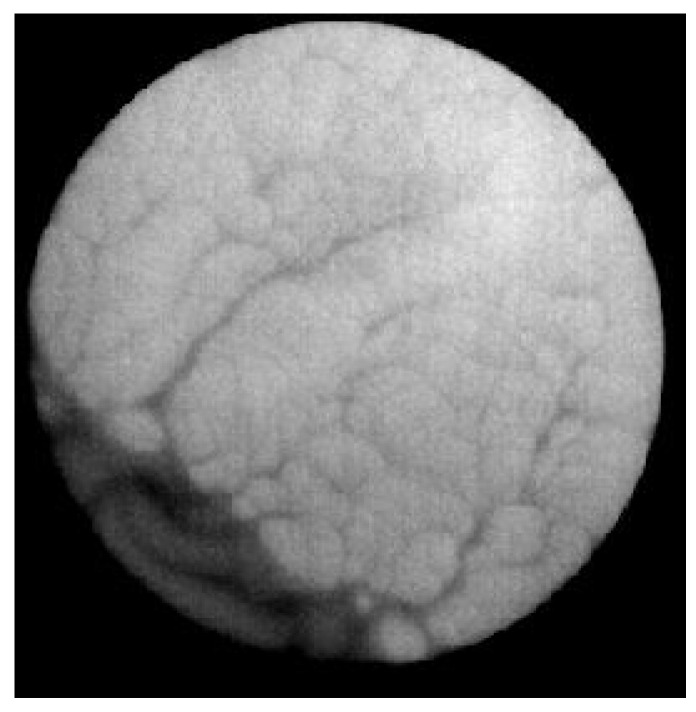
A representative image of a Petri dish with a surface film formed during cooling. The film is the dark part of the image. The lighter part of the image shows the area free of film.

A total of 50 mL of purified water was poured into each of 4 Petri dishes, which were placed in a microwave oven and heated for 90 s at 500 W to a temperature of 53–70 °C. To check the temperature of the solution, the dishes were put into the box and placed so that the distance between the adjacent dishes as well as between the dishes and the box walls was at least 3 cm. The lids were removed from the dishes, and the focus was adjusted. Then, the sequence of thermograms for purified water was recorded, and the proportion of free surface was assessed at 40 °C; it had to be ≥0.8. The total surface area of the sample was limited by the edge of a Petri dish and was characterized by two visible parameters: a solution surface with larger eddies (Rayleigh convection) and smaller eddies (Marangoni convection). Figure 5 shows a photo of a Petri dish containing a purified water sample, where the near-surface layer of a heterogeneous aqueous solution can be clearly seen. 

As discussed above, changes in the properties of the near-surface layer are likely to be associated exactly with a shift in equilibrium between these two dynamic parameters, which, on the one hand, are unique characteristics of the object studied but, most importantly, can affect the equilibrium distribution of the object at a temperature set.

### 3.6. Sample Preparation

S1 solution was obtained when 24.75 mL of purified water and 0.25 mL of the test sample were added to a 40 mL vial, then mixed using a vortex mixer at a speed of 3000 rpm for 5 s; S2 solution was obtained when 2.5 mL of S1 solution and 22.5 mL of purified water were added into a clean 40 mL vial, mixed using a vortex mixer for 5 s at a speed of 3000 rpm; 22 mL was taken from the S2 solution and poured into a clean 250 mL vial; 198 mL of purified water was added, stirred by hand in a circular motion for 5 s from the moment of vortex formation inside the vial.

### 3.7. Analysis

Four aliquots (50 mL each) of the same test sample were poured into Petri dishes, covered with lids, and heated in a microwave oven for 1 min 30 s at 500 W to a temperature of 53–70 °C. To check the temperature of the solution, the 4 dishes were put in the measuring box and placed so that the distance between the adjacent dishes as well as between the dishes and the box walls was at least 3 cm. The lids were removed from the dishes, the least heated Petri dish was cooled to 53 °C and a sequence of thermograms was recorded for all 4 dishes simultaneously. Then, Petri dishes were removed from the measuring box, and, before recording the thermograms of subsequent samples, it was checked that the temperature inside the box equalized (after testing of the samples, heated areas are visible at the bottom of the box). The thermograms with the temperature value were obtained at each point of the frame at a time.

### 3.8. Repeatability, Intermediate Precision and Specificity

These parameters were assessed in accordance with the ICH requirements [18]. Briefly, repeatability expresses the precision under the same operating conditions over a short interval of time. To assess the repeatability, 7 replicates of the same test sample were tested because a minimum of 6 measurements is required for validation. 

Intermediate precision generally expresses within-laboratory variations: different days, different analysts, different equipment, etc. The extent to which intermediate precision should be established depends on the circumstances under which the procedure is intended to be used, and typical variations to be studied include days, analysts, equipment, etc. In this study, the factors of day and operator were analyzed. The analysis was conducted for 12 days by 2 different operators (6 experiments each).

Specificity is the ability to assess the analyte unequivocally in the presence of components that may be expected to be present (like buffer in the antibody solution in our case). Suitable identification tests should be able to discriminate between compounds of closely related structures that are likely to be present. The influence of TPA on another molecule and technologically processed buffer was studied during this work.

### 3.9. Statistical Analysis

Statistical data processing was carried out using a script in Python of at least version 3.7 (Python Software Foundation, Wilmington, DE, USA). The number of sequences and the position of the dishes in the frame were determined. A film-free area was detected for the first and last frames. The film was defined as an area with a lower temperature. For the remaining frames, film-free areas were determined using an interpolation model corresponding to the dish.

Since the boundaries of the surface of the total area are determined by Petri dishes and always constitute two boundaries between these parts, the calculation of both the area free of the film and the area of the film itself can be applied to the same extent. To detect the outliers within each sample, linear trend coefficients k and b were determined for each replicate. Outliers were detected using the DBSCAN coefficient clustering algorithm. Briefly, for each sample, a linear model was approximated for each replicate, based on which the coefficients of the model were determined. Then, using the DBSCAN algorithm, the deviating observations were determined using the coefficients k and b. Observations that deviated by both coefficients were defined as outliers. The function of calculating the percentage difference was indicated as a distance metric. The outliers found for both coefficients were combined.

To compare the samples, the replicates in the temperature range of 30–45 °C, which were not outliers, were averaged. A logarithmic model was built, where the film area was used as a dependent variable; the logarithm of temperature, and sample (as well as all their combinations) were used as independent variables. Based on the models obtained, the mean near-surface film area (*S*) was calculated. The *S* values obtained for the samples were compared using the Tukey test. In the case of a significant difference (*p* < 0.1) in the formation of near-surface film for the solution of the test and control samples, the difference coefficient of near-surface film formation in solution (∆*S*) (2) was additionally calculated:

(2) $$∆S=\left|\frac{S_{Test\;sample}-S_{control}}{S_{contol}}\right|\times 100\%$$

The difference coefficient of the near-surface film formation in the solution of the test sample relative to the control sample must be at least 5% (the statistical significance of the differences corresponds to *p* ≤ 0.1). During the analysis of data from different replicates of the same sample or different days and operators, the observations at temperatures from 30 to 45 °C were adjusted for each group using linear regression (the dependence of the film area on the logarithm of the temperature and the group). After linear regression, post hoc analysis was performed using Tukey’s test to compare groups at the mean temperature range. The data represent conditions of normality and homogeneity of variances. The data with 90% confidence intervals of the difference between the placebo group and the drug group were presented for an intermediate precision experiment performed for 12 days. The inclusion of a confidence interval of zero indicates the absence of statistically significant differences between the placebo and drug groups at a significance level of *p* < 0.1; the exclusion means the statistically significant differences between the placebo and drug groups (*p* < 0.1), respectively. 

## 4. Conclusions

Since the thermographic analysis can evaluate changes in the physical-chemical properties of a solution, which are based on changes in the film formation process, it can be widely applied not only to identify impurities [26], but also to control the quality of various solutions. This method has proven to be a simple and reproducible technique that can be used to assess the identity of TPA-based drugs, regardless of the dosage form. When the difference coefficient of the near-surface formation in the solution of the test sample relative to the control sample is 5% or more, the identity of the test sample must be considered as proved. The validation of this technique as an identity test has been performed and included assessments of specificity versus technologically processed buffer, repeatability for seven replicates, intermediate precision for 12 days, and two operators. 

This technology has become an innovative approach for identification of the TPA-based drugs in liquid dosage form. At the moment, this technique has been validated for the analysis of various dosage forms of TP antibodies to several molecules, but since this approach makes it possible to detect changes in the physical-chemical properties of an aqueous solution in most cases regardless of the nature of the agent changing these properties, the application of this technique for a specific task can be quite wide.

## Data Availability

Data are contained within the article and Supplementary Material.

## Supplementary Materials

The following supporting information can be downloaded at: https://www.mdpi.com/article/10.3390/molecules29184309/s1. Figure S1. An example of images of a Petri dish with a surface film formed during cooling. The film is the dark part of the image. The lighter part of the image shows the area free of film. (A) TPA to S100B; (B) TPA to IFNγ; (C) Placebo.
