# Supplementary material for: Application of High-Resolution Infrared Thermography to Study the Effects of Technologically Processed Antibodies on the Near-Surface Layer of Aqueous Solutions
Source: Molecules. 2024 Sep 11;29(18):4309. doi: 10.3390/molecules29184309 (PMC11434169; doi:10.3390/molecules29184309)
Supplement: Supplementary file 1 [file molecules-29-04309-s001.zip › molecules-3113397-supplementary.pdf]

Supplementary materials

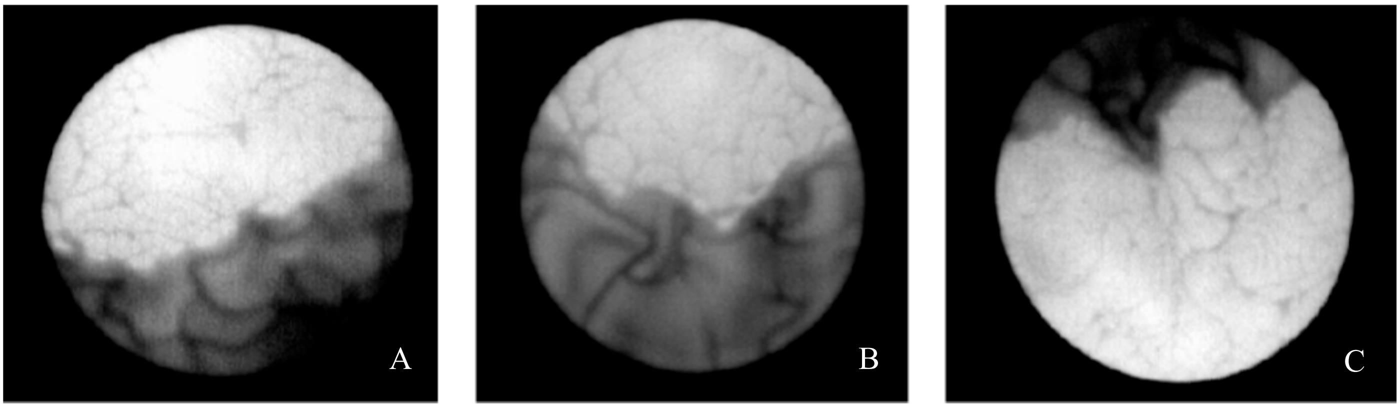

Figure S1. An example of images of a Petri dish with a surface film formed during cooling. The film is the dark part of the image. The lighter part of the image shows the area free of film. A- TPA to S100B; B-TPA to IFNγ; C- Placebo.
